# Use of Physiologically-Based Kinetics Modelling to Reliably Predict Internal Concentrations of the UV Filter, Homosalate, After Repeated Oral and Topical Application

**DOI:** 10.3389/fphar.2021.802514

**Published:** 2022-01-04

**Authors:** Abdulkarim Najjar, Andreas Schepky, Christopher-Tilman Krueger, Matthew Dent, Sophie Cable, Hequn Li, Sebastien Grégoire, Laurene Roussel, Audrey Noel-Voisin, Nicola J. Hewitt, Estefania Cardamone

**Affiliations:** ^1^ Beiersdorf AG, Hamburg, Germany; ^2^ Unilever Safety and Environmental Assurance Centre, Colworth Science Park, Sharnbrook, United Kingdom; ^3^ L'Oréal, Research and Innovation, Aulnay sous Bois, France; ^4^ Cosmetics Europe, Auderghem, Belgium

**Keywords:** homosalate, physiologically-based kinetics models, UV filter, plasma concentration, dermal application

## Abstract

Ethical and legal considerations have led to increased use of non-animal methods to evaluate the safety of chemicals for human use. We describe the development and qualification of a physiologically-based kinetics (PBK) model for the cosmetic UV filter ingredient, homosalate, to support its safety without the need of generating further animal data. The intravenous (IV) rat PBK model, using PK-Sim^®^, was developed and validated using legacy *in vivo* data generated prior to the 2013 EU animal-testing ban. Input data included literature or predicted physicochemical and pharmacokinetic properties. The refined IV rat PBK model was subject to sensitivity analysis to identify homosalate-specific sensitive parameters impacting the prediction of C_max_ (more sensitive than AUC_(0-∞)_). These were then considered, together with population modeling, to calculate the confidence interval (CI) 95% C_max_ and AUC_(0-∞)_. Final model parameters were established by visual inspection of the simulations and biological plausibility. The IV rat model was extrapolated to oral administration, and used to estimate internal exposures to doses tested in an oral repeated dose toxicity study. Next, a human PBK dermal model was developed using measured human *in vitro* ADME data and a module to represent the dermal route. Model performance was confirmed by comparing predicted and measured values from a US-FDA clinical trial (Identifier: NCT03582215, https://clinicaltrials.gov/). Final exposure estimations were obtained in a virtual population and considering the *in vitro* and input parameter uncertainty. This model was then used to estimate the C_max_ and AUC_(0–24 h)_ of homosalate according to consumer use in a sunscreen. The developed rat and human PBK models had a good biological basis and reproduced *in vivo* legacy rat and human clinical kinetics data. They also complied with the most recent WHO and OECD recommendations for assessing the confidence level. In conclusion, we have developed a PBK model which predicted reasonably well the internal exposure of homosalate according to different exposure scenarios with a medium to high level of confidence. In the absence of *in vivo* data, such human PBK models will be the heart of future completely non-animal risk assessments; therefore, valid approaches will be key in gaining their regulatory acceptance.

Clinical Trial Registration: https://clinicaltrials.gov/, identifier, NCT03582215

## Introduction

All chemicals should be assessed for potential toxicity to humans before they are used in products. Traditionally, this has been achieved using standardized animal studies for evaluation of local and systemic effects. These are used to identify adverse effects, target organs, as well as the dose below which no adverse effects are observed (the no observed adverse effect level [NOAEL]). The latter is used as a point of departure (PoD) which can be compared with dose levels experienced by humans through use of the product. However, ethical and legal considerations have led to a change in paradigm towards non-animal methods to evaluate the safety of chemicals for human use. This change is particularly important for cosmetics ingredients, for which animal testing has been banned since March 2013 (Eu, 2009), although non-animal approaches are increasingly of interest for other sectors, such as industrial chemicals and environmental contaminants (Thomas et al., 2019).

The availability of toxico/pharmacokinetics (TK/PK) data greatly increases confidence in risk assessment decisions and can also be valuable in supporting the replacement, reduction and refinement of animal use (3Rs) (Creton et al., 2009). In the absence of animal or human kinetics data, the extrapolation of external doses (in mg/kg/day) to internal dose metrics (C_max_, AUC or concentration at steady state (C_ss_)) must be predicted. Therefore, physiologically-based kinetics (PBK) models are important tools in safety assessment. These are mathematical models used to quantify and predict the absorption, distribution, metabolism and excretion of a chemical following exposure. They are composed of interconnected compartments representing various tissues/organs described by mass balance differential equations that are solved to predict the amount of chemical in each compartment over time (Gerlowski and Jain, 1983). PBK models typically rely on three types of parameters; physiological (e.g., tissue volumes, blood flows), physicochemical (e.g., octanol:water partitioning, vapor pressure, water solubility), and biochemical (e.g., absorption rates, metabolism, clearances). The necessary physiological parameters for several species (mouse, rat, dog and human) are available in the literature (Brown et al., 1997). Tissue:blood partition coefficients for a chemical can be estimated using quantitative structure-property relationships (Peyret et al., 2010), while the clearance of the chemical in different species can be determined using *in vitro* studies with hepatocytes or cellular fractions and incorporated into the PBK model using *in vitro* to *in vivo* extrapolation (Yoon et al., 2012). This modelling approach allows internal concentrations resulting from external exposures to be predicted, allowing comparisons including across species and exposure routes (Clewell and Andersen, 1985).

Guidelines for the application, use, and reporting of PBK models for drugs and chemicals have been published by the FDA and EMA (Ema, 2016; Us fda, 2018). Guidance documents are also available from the Organization for Economic Co-operation and Development (OECD) and World Health Organization (WHO) for the characterization and application of PBK models in the risk assessment of chemicals (WHO, 2010; OECD, 2021), with the aim to harmonize and facilitate their use by chemical developers and regulators. This is particularly important for the cosmetics industry, which fully supports the animal testing ban under the European Union Cosmetics Products Regulation and has decades of commitment in promoting the use of alternatives to animal testing for safety assessment of cosmetics. Indeed, the ambition of Cosmetics Europe (the European cosmetics industry trade association, https://cosmeticseurope.eu/) is to develop non-animal tools and approaches (Desprez et al., 2018). However, historical pre-ban animal toxicology data exist for many cosmetic ingredients, and an important principle in risk assessment is that all available relevant data need to be taken into account when arriving at a safety decision. Such historical data exist for the UV filter homosalate, an ester of salicylic acid and a key cosmetic ingredient in sunscreens. This ingredient provides consumers with protection from the Sun’s [UVA/B] exposure and thus plays a key role in the protection against skin cancer caused by UV exposure. Here, we describe the development and qualification of PBK models for homosalate, which were built to put exposures in an historical animal study into context and substantiate the human safety evaluation without generating further animal data. The aim of this work was to use the PBK model of homosalate to perform route-to-route and inter-species extrapolations to translate both the oral exposures from a historical rat study, and dermal exposures in consumers using sunscreen to internal dose metrics. This could help replace default assessment factors with more specific, substance-derived factors and exclude or refine a toxicokinetic factor in the calculation of margins of safety (SCCS, 2021).

## Methods

### Step 1: Development of the Rat PBK Model

#### Physiological and Physicochemical Properties

PBK modelling was conducted using PK-Sim^®^, OSP Version 9.1 (PK-Sim and MoBi (Bayer Technology Services, Leverkusen, Germany: http://open-systemspharmacology.org) (Willmann et al., 2003; Willmann et al., 2005; Willmann et al., 2007; Thelen et al., 2011). The general concept of building a PBK model previously described by Kuepfer et al. (Kuepfer et al., 2016) was implemented in the current study. The input parameters describing physicochemical properties of homosalate are shown in Table 1. The rat physiological parameters were scaled from the mean body weight of 0.23 kg, age 40 weeks, and a glomerular filtration rate (GFR) of 57 ml/ min/100 g organ (Davies and Morris, 1993).

**TABLE 1:** Physicochemical properties of homosalate [taken from the REACH dossier (ECHA 2020)].

Property	Value
Log P_OW_	6.34 at 40°C
Boiling point	295.1°C at 101.3 kPa
Melting point	< -20°C at 101.3 kPa
Vapor pressure	0.015 Pa at 25°C
Molecular weight	262.344
Water solubility	0.4 mg/ lL at 25°C
pKa	8.1 ± 0.3 at 20°C
Relative density at 20°C relative to water at 4°C	1.0512 (1.050–1.053)

#### Parameter Identification

The base mean model in rats was built using animal data to identify an appropriate structure to describe the plasma kinetics of a single intravenous (IV) bolus dose of 0.5 mg/ kg homosalate reported by Kim et al. (Kim et al., 2014). WebPlotDigitizer-4.3 software (https://automeris.io/WebPlotDigitizer/) was used to extract data from plots presented in the paper. Unknown parameters were identified using the Parameter Identification Module in PK-Sim® and using the Monte-Carlo algorithm. The parameter identification function in PK-Sim was used to optimize the model input parameters, using available kinetics data. The final model parameters were established by visual inspection of the resulting description of data and biological plausibility.

The parameters used for rat model building are summarized in Table 2. The distribution process of homosalate was according to the partition coefficient calculation by Rodgers and Rowland (Rodgers and Rowland, 2006) and the cellular permeability using a charge-dependent model (Schmitt, 2008). Rodgers and Rowland developed two models: one for moderate to strong bases (pKa > 7) and another model for weak, acids and neutral drugs (pKa < 7). The equations predict the steady-state unbound tissue:plasma water partition coefficient, which considers the partitioning of the drug into neutral lipids and phospholipids, the dissolution into tissue water, and the electrostatic interactions with tissue phospholipids. The permeability model developed by Schmitt et al. is calculated from the physicochemical properties and considered the degree of dissociation of acids and bases assuming that the permeabilities for charged species are significantly smaller than for neutral species.

**TABLE 2:** Homosalate parameters used for rat and human model building. Values were obtained from human based *in vitro* assays from the literature, collected from the SCCS report on homosalate 2007 (SCCP, 2007) or newly generated data (unpublished). NA = not applicable.

Property	Rat	Human
Dermal bioavailability		
SCCS report	NA	2% (SCCP, 2007)
CRL Dermal penetration	NA	3.86% (Finlayson, 2021)
CRL Dermal penetration plus 1 SD	NA	5.3% (Finlayson, 2021)
Oral bioavailability		
A Conservative approach	50% (assumption, SCCS default) (adjusted Fa = 81%)	NA
Model-based estimation	Fa = 100%, Fb = 83%	NA
Distribution		
Tissue:plasma partition coefficient	Rodgers and Rowland (Rodgers and Rowland, 2006)	Rodgers and Rowland (Rodgers and Rowland, 2006)
Cellular permeabilities	Charge dependent (Schmitt, 2008)	Charge dependent (Schmitt, 2008)
Fraction unbound (Fu)	2 ± 0.2% (*in silico*)	2 ± 0.2% (*in vitro*)
Elimination		
Total plasma clearance (CLs)	6 L/ h/ kg (Kim et al., 2014)	NA
CL_int, liver_ (primary human hepatocytes)	NA	59.6 ± 2.7 μL min^−1^.10^6^ cells^−1^
*In vitro* half-life (primary human hepatocytes)	NA	11.64 ± 0.53 min
Transporters Substrate	No (*in silico*)	No (*in silico*)

These were selected after testing the available organ-plasma partition coefficient and cell permeability calculation methods built in PK-Sim 9.1. Homosalate fraction unbound (Fu) was initially set at 2.0 ± 0.2%, which was determined from *in silico* and *in vitro* measurements. The contribution of transporters to the distribution of homosalate was evaluated using two *in silico* tools: a substrate classification model provided by SimulationPlus and SwissADME (Daina et al., 2017). These indicated (with a high probability) that homosalate was not a transporter substrate (see Supplementary Materials); therefore, the contribution of these was excluded from the model. Since the focus of this work was on the parent compound, none of the homosalate metabolites were modeled. The elimination rate, represented as total plasma clearance (CLs) of 6 L/ h/ kg, was taken from the *in vivo* rat study (Kim et al., 2014).

#### Sensitivity Analysis, and Uncertainty Calculations

The refined IV rat PBK model was subject to sensitivity analysis relating to organism- and homosalate-specific input parameters using PK-Sim, which identifies a set of variables that impact the estimated kinetics. The sensitivity analysis was conducted, according to PK-Sim (PK-Sim Sensitivity Analysis, 2021) in five steps, with a variation range of 10%/step. The sensitivity for the Pharmacokinetics Parameter = PKj to an input parameter = [pi] was then calculated as the ratio of the relative change of that Pharmacokinetics Parameter [ = (ΔPKj)/PKj ] and the relative variation of the input parameter [ = (Δpi)/pi ]:

$$Sij=\;\frac{\Delta PKj}{\Delta Pi}\;.\;\frac{Pi}{PKj}$$

The sensitivities are dimensionless quantities calculated as the average of several sensitivities based on different variations. Sensitivity analysis results are presented, with sensitivities according to the WHO guidelines (WHO, 2010) as high (absolute value ≥ 0.5), medium (absolute value ≥ 0.2 but less than 0.5) or low (absolute value ≥ 0.1 but < 0.2); parameters with sensitivities < 0.1 are not listed.

Uncertainty expressed by the standard deviation (SD) of the kinetics parameters was considered in the confidence interval CI (5–95)%, such that the influencing parameters were ranked by the SD around the mean of the homosalate-specific parameters. The uncertainty of the *in vitro* generated inputs was also based on the SD. The variations in the physiology and anatomy were considered through the population modeling, where a virtual population was built to scale those parameters.

Uncertainty of the kinetics parameters was expressed as the confidence interval CI (5–95)%, such that the influencing parameters were ranked by the standard deviation (SD) around the mean of the homosalate-specific parameters. The uncertainty of the *in vitro* generated inputs was also based on the SD. The variations in the physiology and anatomy were considered through the population modeling.

To evaluate the performance of the refined model, it was also used to estimate the plasma concentration of the additional IV doses reported in the study by Kim et al. (Kim et al., 2014).

#### Development of the Oral Route Rat PBK Model and Performance

The IV rat model was extrapolated to oral administration in order to convert the doses of 60, 120, 300, and 750 mg/ kg/ day tested in a repeated dose toxicity study in rats (conducted before March 2013, reported in the Scientific Committee on Consumer Safety (SCCS) dossier (SCCS, 2020)) to an internal plasma concentration. The parameters used for rat model building are summarized in Table 2. A model-based approach was used to calculate the intestinal permeability coefficients based on the molecular weight and membrane affinity (Thelen et al., 2011), implemented in PK-Sim 9.1. The refined oral rat PBK model was subject to sensitivity analysis as described above. The oral absorption (Fa) was estimated using *in silico* modelling and was predicted to be almost complete, while, the oral bioavailability (Fb) was predicted to be 83%, which might be lower than Fa due to the first-pass effect of liver metabolism.

Doses tested in a repeated-dose study according to the OECD 422 Test Guideline were simulated to estimate the C_max_ and the area under the curve (AUC_(0–24)_) for each dose. AUC_(0–24)_ values were estimated on the last dosing day (Day 49). The uncertainty of the influencing parameters, including the calculated intestinal permeability, was evaluated according to the C_max_ CI (5–95)%.

### Step 2: Development of the Human PBK Dermal Model

An interspecies extrapolation was conducted by developing the rat model to a human PBK dermal model. The oral administration is not relevant for the human PBK model; therefore, only dermal exposure was considered as a route of exposure.

#### Physiological Properties and Spatial Structure

The spatial structures of the human and rat whole-body models are almost identical, apart from two additional compartments (organs: saliva and gallbladder) in the human model. The anthropometric (height, weight) and physiological parameters in human-adults and rats (e.g., blood flow, organ volumes, binding protein concentrations, hematocrit, cardiac output) were from the literature (Mordenti, 1986; Davies and Morris, 1993; Edginton et al., 2006) or were default values in PK-Sim^®^. The European human individual was characterized by several parameters representing the mean values of age (30 years), body weight (60 kg), and height (163 cm), BMI (22.58 kg/ m^2^), body surface area (BSA, 1.65 m^2^) and GFR (107.44 ml/ min) (taken from Valentin (2002)). The parameters used for human model building are summarized in Table 2. The same mathematical equations for cellular permeability and Fu established in the rat PBK model were used for the human PBK model. The liver was assumed to be the only site of metabolism and the elimination rate of 59.6 ± 2.7 μL min^−1^.10^6^ hepatocytes^−1^ was extrapolated to total hepatic clearance using the IVIVE calculation in PK-Sim 9.1. Based on the Extended Clearance Classification System (ECCS) classification (Varma et al., 2015) and the high plasma binding of homosalate, renal clearance was considered to be negligible and thus was set to zero as a conservative assumption.

#### Dermal Model

The generic human whole-body model was extended with a dermal model for dermal delivery published by (Dancik et al., 2013). This model assumes the skin is a multilayered slab into which compounds can diffuse. Each slab layer corresponds to a skin layer (stratum corneum, viable epidermis and dermis), each of which exhibit diffusion parameters according to its physical and chemical properties. The skin model was built in MoBi (Bayer Technology Services, Leverkusen, Germany (Hamadeh et al., 2019)). The skin permeation model has an air compartment, surface pool compartments, vehicle compartment, skin compartment, and *in vivo* link compartment. The skin compartment consists of three sub-compartments representing the skin layers: stratum corneum sub-compartment, epidermis sub-compartment, dermis sub-compartment. Each of these skin layer sub-compartments comprises four sub-sub-compartments, each of which is composed of ten sub-layers. Dermal clearance in *in vivo* simulations of homosalate undergoes passive transport from all sub-layers of the skin layers into the bloodstream. The diffusion of homosalate through the skin sub-layers was simulated using the methods of finite differences and Fick´s law (Fick, 1855).

Several *in vitro* studies reported dermal penetration of homosalate in frozen non-viable human skin ranged from 1.4 to 3.86%, which was assumed to be due to differences in the formulations used. In these studies, dermal penetration was taken from the SCCS opinion (2% (SCCS, 2020)) and from Finlayson (a mean of 3.86% and the mean plus one SD, 5.3%) to estimate the kinetics parameters following the dermal exposure. Several dermal models were built to simulate the different dermal penetration obtained by the *in vitro* investigations. The main parameters used in the dermal model are listed in the Supplementary Materials. Although *in vitro* assays indicated first-pass metabolism of homosalate in the skin (data not shown), this was excluded from the PBK modeling of dermal exposure. The model assumed that the total penetrated measured amount was homosalate and no metabolites, as a worst-case scenario for systemic exposure to homosalate.

#### Model Performance

The PBK model performance was based on a comparison with results from a clinical trial (Matta et al., 2020). In this study, three US commercially available sunscreen formulations containing 10–15% homosalate and other UV filters under maximal use conditions were topically applied to 12 human volunteers. The sunscreens were applied once on Day 1 and then 4 times (with 2-h intervals) on Day 2 through Day 4, and the plasma concentrations were measured at various time points. The non-aerosol spray containing 10% homosalate was selected as a more conservative comparison compared to the pump spray since it resulted in higher plasma concentrations (17.9 ng/ ml compared to 13.9 ng/ ml, respectively). Homosalate pump spray was applied at 2 mg/ cm^2^ to 75% of the body surface area. The actual homosalate dose applied to 19000 cm^2^ was 150 μg/ cm^2^.

### Step 3: Simulation of Kinetics Following Administration to Rats and Human Virtual Populations

The developed PBK models were used to simulate plasma concentration to homosalate after repeated oral exposure of all the doses administered to rats and after repeated dermal as sunscreen lotion applied on the whole body of humans. The oral dosing to rats was according to an OECD 422 (OECD, 1996) compliant repeated dose toxicity study, in which 20, 60 and 120 mg/ kg homosalate was administered daily for 49 days according to the design of the OECD 422 compliant study conducted prior to March 2013 and reported in the REACH dossier (ECHA, 2020; SCCS, 2020). The virtual rat population was for 100 rats, which was generated from the individual mean value by scaling the body weight from 0.185 to 0.275 kg.

In human studies, sunscreen containing 10% homosalate was topically applied to 17500 cm^2^ of skin twice a day (18 g per day, equivalent to 103 μg/ cm^2^/ day, according to the typical usage reported by the SCCS (SCCS, 2021)). The maximum plasma concentrations at CI95% were simulated in a virtual population and considering the uncertainty of the *in vitro* and input parameters. The PBK human model was built for individuals, with the mean values for anatomy and physiology from the European population. Therefore, population PBK modeling was applied to 100 individuals to consider the variations of the individual anatomy and physiology, and the SD of the homosalate-specific sensitive parameters. The age-based population was generated using PK-Sim by scaling the age from 16 to 70, where the body weight ranged from 45 to 100 kg. The corresponding physiology and anatomy parameters were dependently ranged. The normal distribution of input parameters was implemented, along with the Monte-Carlo algorithm for a random-sampling method. The simulation duration was up to 30 days of bi-daily dermal administration. ADME parameters used for human model building are summarized in Table 2. These included intrinsic clearance and half-life values calculated from incubations with cryopreserved primary human hepatocyte (PHH) suspensions over 2-h (according to the method described by Eilstein et al. (2020)). It was not possible to measure plasma protein binding using standard methods such as rapid equilibrium dialysis, ultrafiltration or ultracentrifugation due to the high non-specific binding to plastic and the hydrolysis of homosalate in plasma (data not shown). Therefore, dedicated protocol was used to overcome these issues, whereby plasma protein binding was calculated from a series of incubations of skin S9 in the presence of a range of concentrations of human serum albumin. This protocol is based on that described by Giulinao et al. who measured intrinsic drug clearance in a microsomal stability assay (Giuliano et al., 2005).

## Results

### Step 1: Development of the Rat PBK Model

#### IV Administration

**FIGURE 1:**
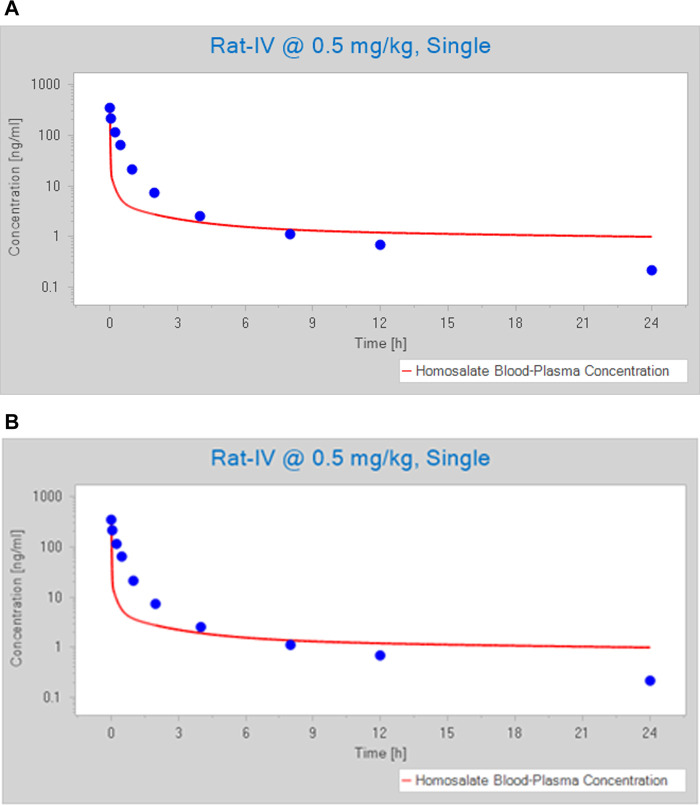
Predicted versus observed (blue circles) concentration-time profile of homosalate after an IV dose of 0.5 mg/ kg. Profiles were generated using **(A)** default values and **(B)** optimized values. The blue circles represent measured values and the red line denoted a simulated profile.

A comparison of the default simulated and measured kinetics profiles of a single dose of 0.5 mg/ kg homosalate is shown in Figure 1A. This shows that the default parameters resulted in a lower C_max_ and AUC_(0–24 h)_ compared to the measured values in rats. Therefore, the automated parameter identification function in PK-Sim was used to optimize several homosalate-specific parameters to fit the observed concentration-time profile of homosalate in rats. These included, Fu and permeability between compartments, which were shown to significantly impact the distribution of homosalate and were therefore optimized using predicted values based on compound properties (e.g., lipophilicity and molecular weight). The optimized Fu was determined to be 1.4%. The optimized permeability values were: interstitial-to-intracellular and intracellular-to-interstitial permeability (4.83 cm/ min); endothelial permeability (1.9 cm/ min); and blood cell-to-plasma partition coefficient (21.28). These optimized values resulted in a concentration-time profile of homosalate that was similar to that measured in rats after IV administration of 0.5 mg/ kg (Figure 1B), with similar mean C_max_ (320 ng/ ml predicted compared to the measured value of 338.3 ± 106.8 ng/ ml) and AUC_(0-∞)_ values (123.1 ng h/ ml predicted compared to the measured value of 113.6 ± 22.9 ng h/ ml).

**FIGURE 2:**
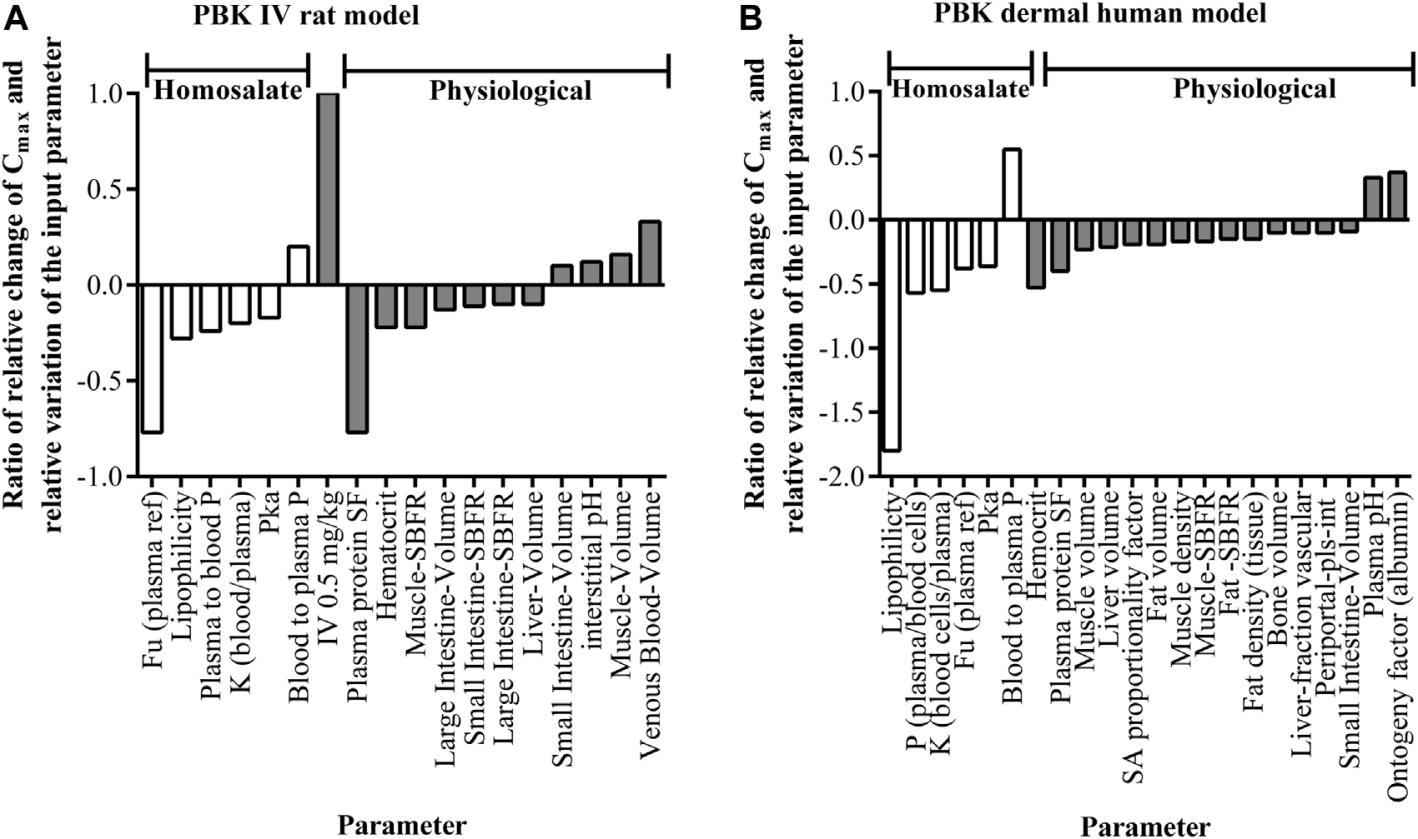
The output of the sensitivity analyses of C_max_ values for the **(A)** rat-IV PBK model (Dose: 0.5 mg/ kg, single) and **(B)** human dermal PBK model (dose 103 μg/ cm^2^, single). The y-axis represents the ratio of the relative change of C_max_ and the relative variation of the input parameter denoted in the x-axis. Parameters with sensitivities less than absolute 0.1 are not listed. Blood to plasma P = blood to plasma permeability; Fu = fraction unbound; K = partition coefficient; Periportal-pls-int P = Periportal-plasma-interstitial permeability; plasma to blood P = plasma to blood permeability; SBFR = specific blood flow rate; SF = scale factor.

A sensitivity analysis was conducted using AUC and C_max_. The outcome indicated that C_max_ was more sensitive to several parameters than AUC (data not shown); therefore, a focus was made on C_max_ to cover remaining influencing parameters. The results of a sensitivity analysis of the homosalate and physiological input parameters impacting the change in C_max_ is shown in Figure 2A. This indicated that the C_max_ is sensitive to several homosalate-specific parameters. The greatest relative change was observed for the Fu, with relative change of −0.77. As with the Fu, four other parameters (lipophilicity, plasma to blood permeability and blood/plasma partition coefficient and pKa) also negatively impacted the C_max_. By contrast, the blood/plasma permeability positively impacted the C_max_ (with a ratio of 0.2). These influencing parameters altered the C_max_ within one SD. Consequently, the estimated kinetics parameter uncertainty was set at the 5^th^ to 95^th^ C_max_ around the mean value, where the SD around the mean defined the range of the influencing parameters.

**FIGURE 3:**
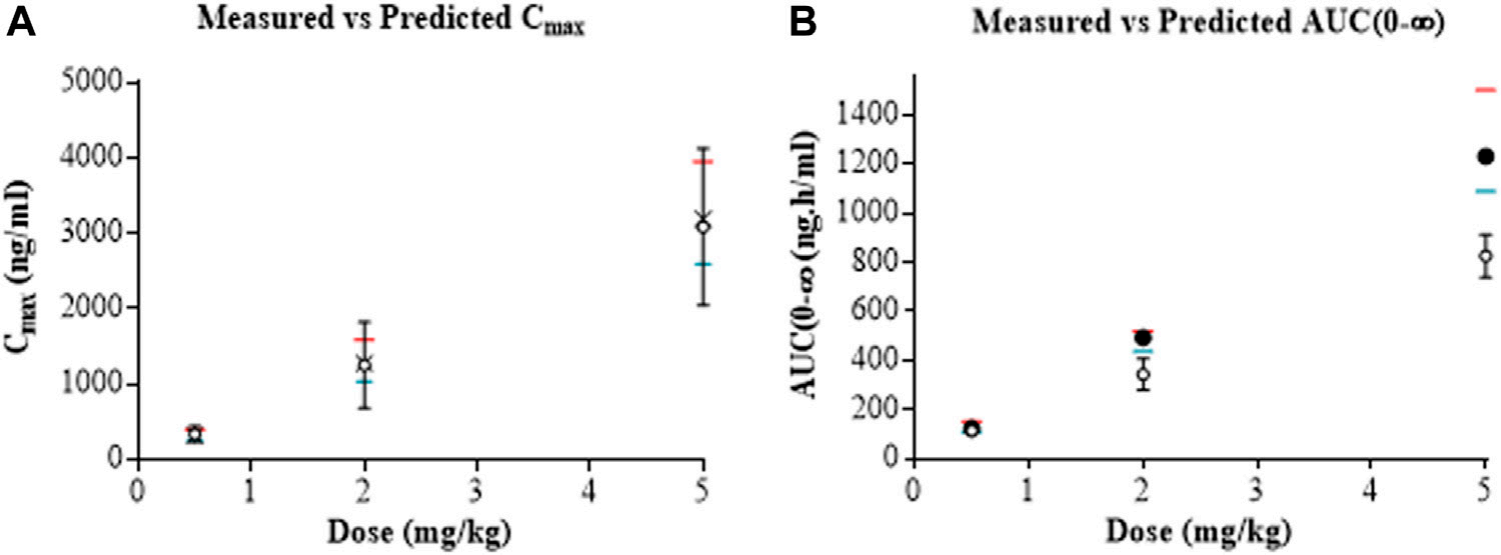
Predicted mean (closed circles) and CI95% (crosses) versus observed (open circles) C_max_ **(A)** and AUC_(0-∞)_ **(B)** of homosalate after IV doses of 0.5, 2 and 5 mg/ kg to rats. Predictions were generated using optimized input values. The CI95% (red lines) and CI5% (blue lines) are also included.

The mean and CI 95% of AUC_(0-∞)_ and C_max_ of the additional doses, 2 and 5 mg/ kg, were also estimated using the optimized model and were shown to be similar to *in vivo* values (shown in Figure 3, along with the comparison of the dose of 0.5 mg/ kg). The simulated kinetics indicated a linear correlation between the AUC_(0-∞)_ and C_max_ with the dose, which was in accordance with *in vivo* findings. These results showed that the model performed well and could be used to extrapolate to the oral route.

#### Oral Administration

The optimized PBK rat model was used to estimate the C_max_ and AUC_(0–24)_ of homosalate after single and repeated oral administration of 60, 120, 300 and 750 mg/ kg/ day to rats. This would enable an understanding of the internal concentration across this dose range and; therefore, provide a more relevant representation of exposure than the applied dose (in mg/kg) to compare with human exposure (SCCS, 2021). The model-based approach used by Thelen et al. (Thelen et al., 2011) to calculate the intestinal permeability coefficients predicted the oral absorption (Fa) to be almost complete (i.e., 100%); however, the oral bioavailability (Fb) was predicted to be 83%, which might be due to a first-pass effect. However, the SCCS recommends a value 50% of the administered dose for chemicals without measured kinetics; therefore, this value was used as a more conservative approach which could be considered to incorporate a higher first-pass effect (since a lower systemic concentration would be linked to any adverse effects). The intestinal permeability was adjusted to result in an oral bioavailability (Fb) of 50%. As a result, the corresponding oral absorption (Fa) was estimated at 81%. Due to the lack of available data, absorption kinetics was kept as default; however, both internal exposure metrics (short term: C_max_; and long term: AUC) were reported and can be implemented for the calculation in the safety assessment. The predicted C_max_ and AUC_(0–24 h)_ values for each of the doses are shown in Table 3.

**TABLE 3:** Predicted kinetics parameters of homosalate in rats after repeated oral administration.

	Rat-oral, bioavailability 50%, mg/kg/day			
	60	120	300	750
C_max_: mean, CI (5–95)%, (ng/ml)	932.4 (769.21–1192)	1837.8 (1512–2339)	4905 (4084–6188.8)	11543 (9581–14545)
AUC_(0–24)_: mean, CI (5–95)%, (ng.h/ml)	16174.05 (13295.64–20953.58)	31979.89 (26443.22–40847.898)	84295 (70787–104639)	200744 (167901–250263)

### Step 2: Development of the Human PBK Dermal Model

A preliminary sensitivity analysis was conducted on the skin penetration model (Dancik et al., 2013) to assess the effect of varying the main model parameters on the cumulative permeant mass (*Q*) that crosses partially hydrated skin over a 24 h period. The parameters tested were the partition coefficients with respect to water and the diffusion coefficients of each skin layer. The physicochemical properties specific to the permeant (homosalate) were used as input into the model, which, as a first step, calculates the nominal partition and diffusion coefficients of the compound. The stratum corneum was identified as the main barrier to homosalate skin penetration, with a negligible contribution of the epidermis and dermis to the overall cumulative mass penetration. Thus, the initial sensitivity analysis demonstrated that the cumulative mass, *Q*, of homosalate crossing the skin was most sensitive to the properties of the stratum corneum. The relevant system-dependent parameters were thus identified as those that comprise the stratum corneum partition coefficient with respect to water (K_sc/w_) and the stratum corneum diffusion coefficient (D_sc_). The derivation of these parameters from the parameters in the model are discussed by Danick et al. (Dancik et al., 2013). The variability in the skin model parameters translates to a significant variation in *Q,* which can impact the whole-body permeant disposition. When default input values were used, the skin absorption was predicted to be 0.6% of the applied dose, which is 3-fold lower than that was considered for a risk assessment (SCCP, 2007). Thus, three pharmacokinetic analyses were therefore conducted: In the first, the parameters of the skin permeation model were set to generate a cumulative mass penetration equal to *Q =* 2.07 μg/ cm^2^/24 h, representing 2% skin penetration of homosalate. In the second and third analysis, the parameters were set to deliver 4 and 5.46 μg/ cm^2^/24 h, representing 3.86 and 5.3% of applied homosalate, respectively. The cumulative amount of homosalate (Q) entering the bloodstream per cm^2^ over 24 h, excluding first-pass skin metabolism, was incorporated using these values, and ranged from 2.07 to 5.46 μg/ cm^2^ (Table 4). The corresponding C_max_ and AUC_(0–24 h)_ values were then estimated by the human whole-body PBK model, which ranged from the mean of 2.38–6.3 ng/ ml and 49.39–130.40 ng h/ ml, respectively. The linear relationship between the C_max_ and the dose, reported in rats, was also observed in the human dermal PBK model.

**TABLE 4:** Impact of the dermal delivery value on the estimated C_max_ of homosalate after a single dermal administration. For each scenario, a single dose of 10% homosalate was applied to the whole-body surface (17500 cm^2^) at 103 μg/ cm^2^/ day. The values for the Day 1 kinetics.

	Dermal delivery (% of the applied dose)		
	2%	3.86%	5.3%
Dermal delivery of the dermal model: cumulative amount (Q) over 24 h (µg/cm^2^)	2.07	4	5.46
C_max_ (ng/ml) mean	2.38	4.63	6.3
C_max_ (ng/ml) CI95%	4.13	8.04	10.92
AUC_(0-24h)_ (ng.h/ml) mean	49.39	95.26	130.40
AUC_(0-24h)_ (ng.h/ml) CI95%	84.75	163.48	223.76

The results of a sensitivity analysis of the impact of homosalate-specific and physiological input parameters on the change in C_max_ in human plasma after topical application is shown in Figure 2B. This indicated that, apart from the organism-specific input parameters, the C_max_ was sensitive to several homosalate-specific parameters. As with the rat PBK model, the Fu negatively impacted the C_max_ (with a ratio of −0.38), although the greatest relative negative change was observed for the lipophilicity, with relative change of −1.8. Three other parameters (plasma/blood cell permeability, blood cells/plasma partition coefficient and pKa) also negatively impacted the C_max_. As for the rat PBK model, the blood/plasma permeability increased the C_max_ (with a ratio of 0.55). The same set of homosalate parameters was therefore used to calculate the uncertainty of C_max_ following dermal exposure (based on the SD of the influencing input parameters). The resulting developed models were applicable for a range of dermal penetration of homosalate from 2 to 5.3%, including uncertainty resulting from formulation effects.

**FIGURE 4:**
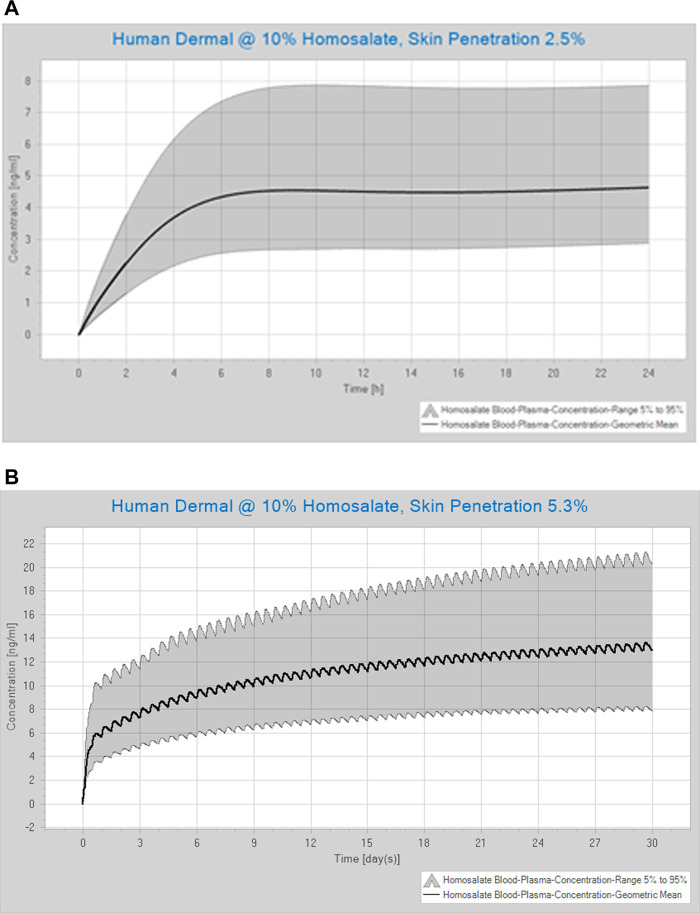
Simulated concentration-time profile of homosalate **(A)** over 24 h after a single application, where the dermal penetration was estimated to be 2.5% (this scenario mimics that tested by Matta et al. (2020)), and **(B)** over 30 days of repeated dermal exposure, where the dermal penetration was estimated to be 5.3% of the applied dose (this scenario mimics the dosing for the safety assessment). Values are shown for the mean and the CI (5–95)%. The application was of 10% homosalate applied to the human whole-body.

The human PBK dermal model performance was evaluated by comparing predicted values with measured values over 24 h from a clinical trial (Matta et al., 2020). When the dermal penetration was set at the lowest value of 2% of the applied dose, the mean C_max_ (3.72 ng/ ml) was comparable to that observed in humans (4.6 ng/ ml). However, the simulated C_max_ CI95% (6.45 ng/ ml) accounted for only ∼73.3% of the clinical C_max_ values (which ranged from 2 to 8.8 ng/ ml). Therefore, the dermal penetration value was adjusted to result in a more comparable C_max_ (mean = 4.55, CI95% = 7.88 ng/ ml), which covered 89.6% of the clinical values (Figure 4A). The corresponding dermal penetration was estimated to be 2.48% of the applied dose. The plasma T_max_ of 10–12 h observed in humans was also predicted by the dermal model.

### Step 3: Simulation of Kinetics Following Administration to Human Virtual Populations

The optimized human PBK dermal model was used to estimate C_max_ and AUC_(0–24)_ values after repeated bi-daily dermal exposure of product containing 10% homosalate for 30 days. The simulated concentration-time profile of homosalate following repeated dermal exposure over 30 days, for which the dermal penetration was estimated to be 5.3% of the applied dose, is shown in Figure 4B. The plasma concentration was predicted to rise rapidly in the first 24 h and then slowly increase after this time. Table 5 shows the impact of using different values for dermal penetration on the internal exposure to homosalate present at 10% in a product. The mean C_max_ ranged from 5.22 ng/ ml using 2.0% of the applied dose to 13.62 ng/ ml when using 5.3% of the applied dose. The mean AUC_(0–24)_ ranged from 119.81 ng h/ ml using 2.0% of the applied dose to 316.49 ng h/ ml when using 5.3% of the applied dose.

**TABLE 5:** Estimated kinetics parameters of homosalate after repeated dermal exposure over 30 days and different dermal penetration potential. For each scenario, 10% homosalate was applied to the whole-body surface (17500 cm^2^) twice a day (18 g cream per day, 103 μg/ cm^2^/ day). The same set of the influencing homosalate-specific input parameters and the population modeling were implemented to calculate the C_max_ uncertainty. The values for the Day 30 kinetics.

	Dermal penetration (% of the applied dose)			
	2.0	2.5	3.86	5.3
C_max_ (mean) (ng/ml)	5.22	6.65	9.94	13.62
C_max_ (CI5-95%) (ng/ml)	3.16–8.17	4.02–10.4	6.01–15.53	8.24–21.27
AUC_(0–24)_: (mean) (ng.h/ml)	119.81	153.02	230.96	316.49
AUC_(0–24)_: (CI5-95%) (ng.h/ml)	72.6–187.09	92.72–238.96	139.92–360.68	191.73–494.26

## Discussion

**FIGURE 5:**
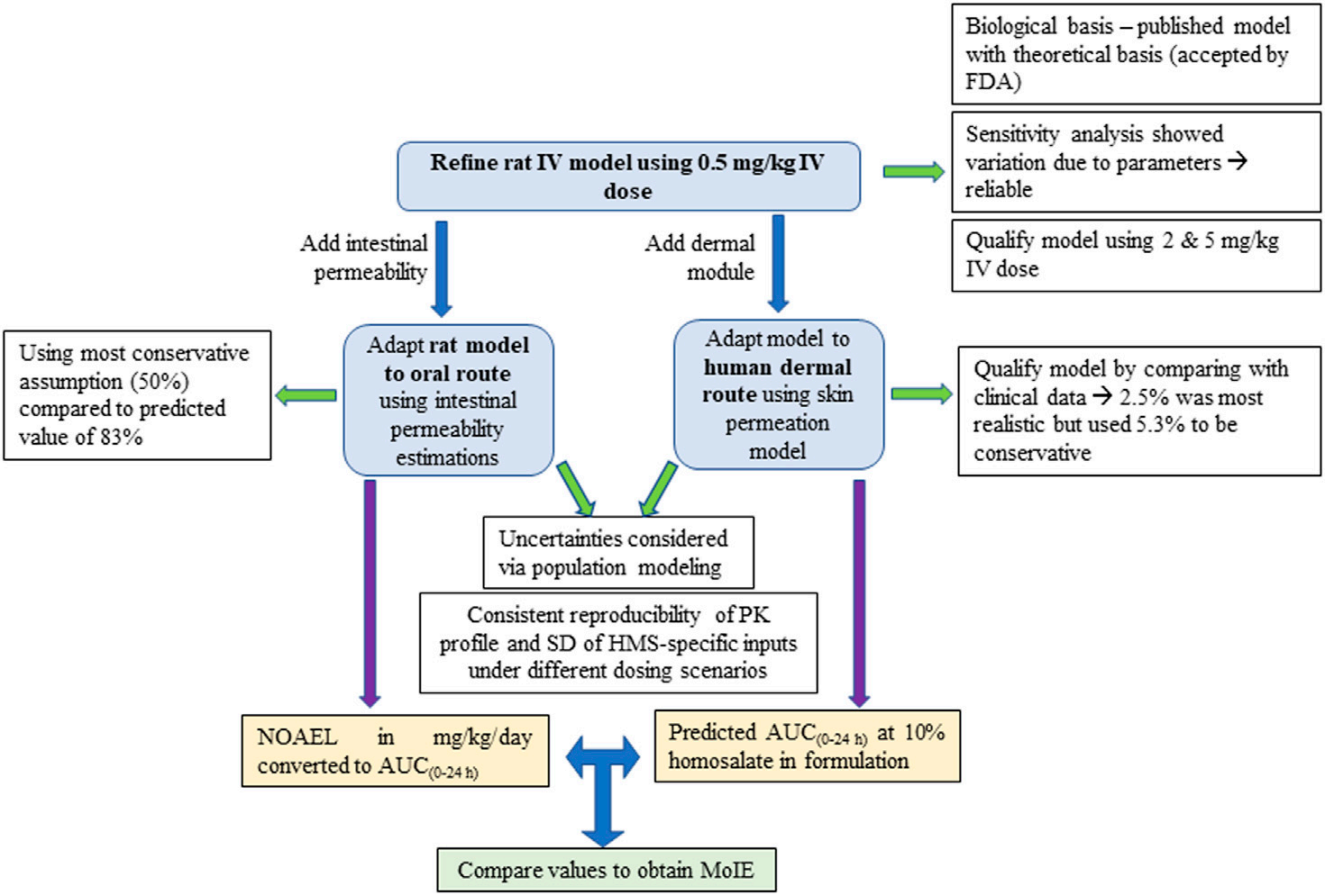
An overview of the development of the PBK models, together with the associated assessments for model qualification and the derivation of the MoIE.

In common with many jurisdictions, the European cosmetics industry fully supports the animal testing ban under the EU Cosmetics Products Regulation. The industry has decades of commitment in promoting the use of alternatives to animal testing for safety assessment of cosmetics and has been at the forefront of the development and use of non-animal testing methods for safety assessment. One approach to allow animal-free decision-making is “Next generation risk assessment” (NGRA), which is an exposure-led and hypothesis-driven tiered workflow designed to prevent harm to humans (Dent et al., 2021). One of the principles underpinning the NGRA of cosmetic ingredients is that it is only conducted following an appraisal of all existing information, which may already be sufficient to make a safety decision. This is an important principle because many cosmetic ingredients have been used safely for decades and it would not be sensible to ignore historical animal data generated prior to the animal testing ban. This is the case for homosalate, which was the subject of historical animal tests which need to be put into context for the overall safety evaluation. One of the critical tools that allows this is PBK modelling. Here, PBK models were built and refined to estimate homosalate plasma concentrations over time, first, in rats after repeated oral exposure to a dose related to a no effect level observed in a toxicity study and, second, in humans following consumer-relevant dermal exposure as a sunscreen to the whole-body. The plasma values (e.g., mean, CI5% or CI95%, depending on the level of conservatism and the interpretation of the safety assessor) can then be compared to calculate a MoIE, which should ideally be above a safety factor of 25 to be considered protective of human health. This safety factor is based on the safety factors described by WHO (World Health Organization, 2005), which account for an overall uncertainty of 25 due to animal to human differences in toxicodynamics (safety factor = 2.5), human variability in toxicodynamics (safety factor = 3.16) and human variability in toxicokinetics (safety factor = 3.16). An overview of the development of the PBK models is shown in Figure 5, along with their associated assessments for model qualification and the derivation of the MoIE.

The PBK models were developed in a stepwise manner. The first step was to build a PBK rat model, which was optimized to simulate the kinetics of a single IV dose of 0.5 mg/kg homosalate administered to rats. While it is understood that the use of animal data is not currently possible in the cosmetics industry, legacy data generated prior to March 2013 can be used if available. Since the experimental work of the rat study reported by Kim et al. was conducted prior to the animal testing ban, it could be used for the purpose of model building and qualification. In the absence of the *in vivo* data, our study showed that the default values of the PK-Sim model would underpredict the internal exposure (AUC_(0–24 h)_ and C_max_) to homosalate. This would be considered a conservative model, since toxicity observed would be linked to a lower plasma concentration that was actually present. The optimization using automated parameter identification function in PK-Sim ensured that a more realistic prediction of plasma concentrations was obtained. The accuracy of the refined model was confirmed by comparing simulated and measured kinetics profiles of two additional IV doses in rats.

The IV model was then adapted to incorporate intestinal permeability to enable the extrapolation of the NOAEL in a repeated oral dose study in rats to a plasma concentration. Two different values for bioavailability were considered but since the value of 50% recommended by the SCCS leads to a lower plasma concentration than the *in silico* derived oral bioavailability value of 83%, the former was incorporated to consider biologically plausible first-pass metabolism and to ensure a more conservative value. This was also considered prudent because measured data were unavailable for comparison with the resulting kinetics profile.

A PBK human dermal model was then developed to estimate blood concentrations following repeated topical exposure of humans to a product containing 10% homosalate. This value can be compared with the plasma concentration at the NOAEL to calculate the MoIE for the risk assessment (using mean values of AUC_(0–24 h)_, see Figure 4). This allows the determination of a safe dose of homosalate based on a systemic exposure-based risk assessment rather than an external exposure-based risk assessment. The refined dermal model was suitable for varying extents of dermal penetration, although the value that best reflected the plasma concentrations measured in humans was 2.5% of the applied dose. When considering a risk assessment, the highest value that considers formulation effects (i.e., 5.3% of the applied dose) can be considered to be the most conservative.

The WHO and OECD Test Guidelines require a PBK model to be valid and applicable to the purpose of its use. The model validity (i.e., reliability and relevance) is based on several factors, including (1) a biological basis of the model structure and parameters, (2) a theoretical basis of the model equations, (3) reliability and relevance of input parameters, and (4) the sensitivity of the output to input parameters (WHO, 2010; OECD, 2021). For the first two requirements, the PK-Sim is an ideal platform with a high level of confidence, since it is an open-source model for which the codes, equations parameters, structure and assumptions are freely available. It is also accepted by the FDA for waiving of clinical trials (Zhuang and Lu, 2016). Several assumptions were implemented in the PBK model e.g., it assumes perfusion rate/blood flow limited kinetics for homosalate within the organs and tissues, which describes the kinetics for small lipophilic molecules. The developed model consistently reproduced the general kinetics of the parent molecule under various conditions i.e., different doses, routes of exposure (IV, dermal exposure), ultimately simulating kinetics profiles for the intended applications in rats and humans.

An important requirement in building a PBK model to confirm its validity is to subject it to a sensitivity analysis to identify the parameters which markedly influence the predictions. A model is considered to be reliable when a small change in a parameter value leads to changes in predictions of a dose metric that are less than the variation expected from its experimental measurement (WHO, 2010; OECD, 2021). The PBK model here was shown to be sensitive to parameter changes, whereby sensitivity analysis identified several homosalate-specific parameters (calculated partition coefficient and permeability blood cells/plasma) which influenced the C_max_ within one SD of the mean. The uncertainty of the *in vitro* measured parameters (Fu and metabolic clearance) was considered within the SD. The skin penetration model was optimized to recapitulate *in vitro* data and, due to the variation of values of homosalate dermal penetration (2–5.3% of the applied dose, presumably due to formulation effects and the inherent variability between skin penetration studies using different donors), several skin models were built to address dermal penetration uncertainty. The uncertainty calculated in the current study covers both true uncertainty (homosalate-specific parameters) and variability (population variability). Uncertainties in the whole-body PBK model were considered via population modeling, where a virtual population was built to cover variations in the anatomy and physiology of humans and rats. This covered the variation of the organ volumes, organ composition, tissue and body fluid physiology and blood flow rates.

The degree of confidence in the predictions of dose metrics by a PBK model depends upon how well it has been tested against measured data from a variety of dosing scenarios (WHO, 2010). The level of confidence in the model described here was considered to be medium-to-high for rat kinetics and high for human kinetics, since it was able to simulate the general trend of the concentration-time profile (peaks and decline) of homosalate under different exposure scenarios in humans and rats. This was evident upon visual inspection and, quantitatively, with respect to the plasma concentrations. While this was possible in the current study, PBK models for other compounds may not be possible and would rely on the quality of the *in vitro* data used as input, as well as the use of sensitivity analysis to assess the confidence for a specific purpose (OECD, 2021).

In conclusion, we have developed PBK models which are able to correctly predict the internal exposure of homosalate according to different exposure scenarios. There was a medium-to-high level of confidence in the model output according to the classification defined in guidance documents (WHO, 2010; OECD, 2021) and; therefore, is useful in risk assessment. The process established here may serve as a reference supporting future homosalate PBK studies (e.g., extrapolation across dose-routes, between species, from high to low dose levels, and over various dosing scenarios), in further safety assessments on homosalate exposure. Indeed, in the absence of *in vivo* data, such human PBK models will be the heart of future completely non-animal risk assessments; therefore, valid approaches, as used here, will be key in gaining their regulatory acceptance.

## Data Availability

The raw data supporting the conclusions of this article will be made available by the authors, without undue reservation.

## Abbreviations

ADME, absorption, distribution, metabolism and excretion; BSA, body surface area; Css, concentration at steady state; CI, confidence interval; CLs, total plasma clearance; ECCS, Extended Clearance Classification System; Fa, oral absorption; Fb, oral bioavailability; GFR, glomerular filtration rate; IV, intravenous; MoIE, Margin of Internal Exposure; NOAEL, no observed adverse effect level; OECD, Organization for Economic Co-operation and Development; PBK, physiologically-based kinetics; PoD, point of departure; SCCS, Scientific Committee on Consumer Safety; SVM, Support Vector Machine; TK/PK, toxico/pharmacokinetics; WHO, World Health Organization.

## References

[B1] Brown R. P. Delp M. D. Lindstedt S. L. Rhomberg L. R. Beliles R. P. (1997). Physiological Parameter Values for Physiologically Based Pharmacokinetic Models. Toxicol. Ind. Health 13, 407–484. 10.1177/074823379701300401 9249929

[B2] Clewell H. J. 3rd Andersen M. E. (1985). Risk Assessment Extrapolations and Physiological Modeling. Toxicol. Ind. Health 1, 111–131. 10.1177/074823378500100408 3843496

[B3] Creton S. Billington R. Davies W. Dent M. P. Hawksworth G. M. Parry S. (2009). Application of Toxicokinetics to Improve Chemical Risk Assessment: Implications for the Use of Animals. Regul. Toxicol. Pharmacol. 55, 291–299. 10.1016/j.yrtph.2009.08.001 19665509

[B4] Daina A. Michielin O. Zoete V. (2017). SwissADME: a Free Web Tool to Evaluate Pharmacokinetics, Drug-Likeness and Medicinal Chemistry Friendliness of Small Molecules. Sci. Rep. 7, 42717. 10.1038/srep42717 28256516 PMC5335600

[B5] Dancik Y. Miller M. A. Jaworska J. Kasting G. B. (2013). Design and Performance of a Spreadsheet-Based Model for Estimating Bioavailability of Chemicals from Dermal Exposure. Adv. Drug Deliv. Rev. 65, 221–236. 10.1016/j.addr.2012.01.006 22285584

[B6] Davies B. Morris T. (1993). Physiological Parameters in Laboratory Animals and Humans. Pharm. Res. 10, 1093–1095. 10.1023/a:1018943613122 8378254

[B7] Dent M. P. Vaillancourt E. Thomas R. S. Carmichael P. L. Ouedraogo G. Kojima H. (2021). Paving the Way for Application of Next Generation Risk Assessment to Safety Decision-Making for Cosmetic Ingredients. Regul. Toxicol. Pharmacol. 125, 105026. 10.1016/j.yrtph.2021.105026 34389358 PMC8547713

[B8] Desprez B. Dent M. Keller D. Klaric M. Ouédraogo G. Cubberley R. (2018). A Strategy for Systemic Toxicity Assessment Based on Non-animal Approaches: The Cosmetics Europe Long Range Science Strategy Programme. Toxicol. Vitro 50, 137–146. 10.1016/j.tiv.2018.02.017 29499337

[B9] ECHA (2020). Registration Dossier. Retrieved from: https://echa.europa.eu/en 1/registrationdossier/-/registered-dossier/13246 2020 .

[B10] Edginton A. N. Schmitt W. Willmann S. (2006). Development and Evaluation of a Generic Physiologically Based Pharmacokinetic Model for Children. Clin. Pharmacokinet. 45, 1013–1034. 10.2165/00003088-200645100-00005 16984214

[B11] Eilstein J. Grégoire S. Fabre A. Arbey E. Géniès C. Duplan H. (2020). Use of Human Liver and EpiSkin™ S9 Subcellular Fractions as a Screening Assays to Compare the *In Vitro* Hepatic and Dermal Metabolism of 47 Cosmetics-Relevant Chemicals. J. Appl. Toxicol. 40, 416–433. 10.1002/jat.3914 31912921

[B12] Ema (20162016). European Medicines Agency. Guideline on the Qualification and Reporting of Physiologically Based Pharmacokinetic (PBPK) Modelling and Simulation. Available at: http://www.ema.europa.eu/docs/en_GB/document_library/Scientific_guideline/2016/07/WC500211315.pdf.

[B13] Eu (2009). Consolidated Text: Regulation (EC) No 1223/2009 of the European Parliament and of the Council of 30 November 2009 on Cosmetic Products (Recast) (Text with EEA relevance)Text with EEA Relevance. Available at: http://data.europa.eu/eli/reg/2009/1223/2021-08-23.

[B14] Fick A. (1855). V. On Liquid Diffusion. Lond. Edinb. Dublin Philos. Mag. J. Sci. 10, 30–39. 10.1080/14786445508641925

[B15] Gerlowski L. E. Jain R. K. (1983). Physiologically Based Pharmacokinetic Modeling: Principles and Applications. J. Pharm. Sci. 72, 1103–1127. 10.1002/jps.2600721003 6358460

[B16] Giuliano C. Jairaj M. Zafiu C. M. Laufer R. (2005). Direct Determination of Unbound Intrinsic Drug Clearance in the Microsomal Stability Assay. Drug Metab. Dispos 33, 1319–1324. 10.1124/dmd.105.005033 15951446

[B43] Finlayson Z. (2021). The *In Vitro* Percutaneous Absorption of [14 C] -Homosalate in a Cosmetic Formulation Through Human Split-Thickness Skin according to OECD Guideline 428, SCCS/1358/10. Charles River Laboratories Edinburgh Ltd. Test Facility Study No. 786915.

[B17] Hamadeh A. Sevestre M. Edginton A. (2019). Implementation of Dancik et al (2013) skin permeation model in MoBi. 22 May 2019. [Online]. Available [web site]: https://github.com/Open-Systems-Pharmacology/Skin-permeation-model [Accessed].

[B18] Kim T. H. Shin B. S. Kim K. B. Shin S. W. Seok S. H. Kim M. K. (2014). Percutaneous Absorption, Disposition, and Exposure Assessment of Homosalate, a UV Filtering Agent, in Rats. J. Toxicol. Environ. Health A. 77, 202–213. 10.1080/15287394.2013.861376 24555679

[B19] Kuepfer L. Niederalt C. Wendl T. Schlender J. F. Willmann S. Lippert J. (2016). Applied Concepts in PBPK Modeling: How to Build a PBPK/PD Model. CPT Pharmacometrics Syst. Pharmacol. 5, 516–531. 10.1002/psp4.12134 27653238 PMC5080648

[B20] Matta M. K. Florian J. Zusterzeel R. Pilli N. R. Patel V. Volpe D. A. (2020). Effect of Sunscreen Application on Plasma Concentration of Sunscreen Active Ingredients: A Randomized Clinical Trial. Jama 323, 256–267. 10.1001/jama.2019.20747 31961417 PMC6990686

[B21] Mordenti J. (1986). Man versus Beast: Pharmacokinetic Scaling in Mammals. J. Pharm. Sci. 75, 1028–1040. 10.1002/jps.2600751104 3820096

[B22] OECD (2021). Guidance Document on the Characterisation, Validation and Reporting of Physiologically Based Kinetic (PBK) Models for Regulatory Purposes. Organisation for Economic Co-operation and Development. OECD Series on Testing and Assessment, No. 331, Environment, Health and Safety.

[B23] OECD (1996). Test No. 422: Combined Repeated Dose Toxicity Study with the Reproduction/Developmental Toxicity Screening Test.

[B24] Peyret T. Poulin P. Krishnan K. (2010). A Unified Algorithm for Predicting Partition Coefficients for PBPK Modeling of Drugs and Environmental Chemicals. Toxicol. Appl. Pharmacol. 249, 197–207. 10.1016/j.taap.2010.09.010 20869379

[B25] Pk-Sim Sensitivity Analysis. (2021) Retrieved from [web site]: https://docs.open-systems-pharmacology.org/shared-tools-and-example-workflows/sensitivity-analysis [Accessed].

[B26] Rodgers T. Rowland M. (2006). Physiologically Based Pharmacokinetic Modelling 2: Predicting the Tissue Distribution of Acids, Very Weak Bases, Neutrals and Zwitterions. J. Pharm. Sci. 95, 1238–1257. 10.1002/jps.20502 16639716

[B27] SCCP (2007). Opinion of the SCCP on Homosalate, SCCP/1086/07. Available at: https://ec.europa.eu/health/ph_risk/committees/04_sccp/docs/sccp_o_097.pdf.

[B28] SCCS (2020). SCCS (Scientific Committee on Consumer Safety), Opinion on Homosalate. (CAS No 118-56-9, EC No 204-260-8), preliminary version of 27-28 October 2020, final version of 24-25 June 2021, SCCS/1622/20.

[B29] SCCS (2021). Scientific Committee on Consumer Safety), SCCS Notes of Guidance for the Testing of Cosmetic Ingredients and Their Safety Evaluation 11th Revision, 30–31. March 2021, SCCS/1628/21. 10.1016/j.yrtph.2021.105052 34653552

[B30] Schmitt W. (2008). General Approach for the Calculation of Tissue to Plasma Partition Coefficients. Toxicol. Vitro 22, 457–467. 10.1016/j.tiv.2007.09.010 17981004

[B31] Thelen K. Coboeken K. Willmann S. Burghaus R. Dressman J. B. Lippert J. (2011). Evolution of a Detailed Physiological Model to Simulate the Gastrointestinal Transit and Absorption Process in Humans, Part 1: Oral Solutions. J. Pharm. Sci. 100, 5324–5345. 10.1002/jps.22726 21993815

[B32] Thomas R. S. Bahadori T. Buckley T. J. Cowden J. Deisenroth C. Dionisio K. L. (2019). The Next Generation Blueprint of Computational Toxicology at the U.S. Environmental Protection Agency. Toxicol. Sci. 169, 317–332. 10.1093/toxsci/kfz058 30835285 PMC6542711

[B33] Us fda (2018). Physiologically Based Pharmacokinetic Analyses—Format and Content. Guidance for Industry. Washington, DC: Center for Drug Evaluation and Research.

[B34] Valentin J. (2002). Basic Anatomical and Physiological Data for Use in Radiological protection: Reference Values. A Report of Age- and Gender-Related Differences in the Anatomical and Physiological Characteristics of Reference Individuals. ICRP Publication 89. Ann. ICRP 32, 5–265. 10.1016/s0146-6453(03)00002-2 14506981

[B35] Varma M. V. Steyn S. J. Allerton C. El-Kattan A. F. (2015). Predicting Clearance Mechanism in Drug Discovery: Extended Clearance Classification System (ECCS). Pharm. Res. 32, 3785–3802. 10.1007/s11095-015-1749-4 26155985

[B36] WHO (2010). Project H, No D. Characterization and Application of Physiologically Based Pharmacokinetic Models. IPCS - WHO. Available at: https://apps.who.int/iris/bitstream/handle/10665/44495/9789241500906_eng.pdf .

[B37] Willmann S. Höhn K. Edginton A. Sevestre M. Solodenko J. Weiss W. (2007). Development of a Physiology-Based Whole-Body Population Model for Assessing the Influence of Individual Variability on the Pharmacokinetics of Drugs. J. Pharmacokinet. Pharmacodyn 34, 401–431. 10.1007/s10928-007-9053-5 17431751

[B38] Willmann S. Lippert J. Schmitt W. (2005). From Physicochemistry to Absorption and Distribution: Predictive Mechanistic Modelling and Computational Tools. Expert Opin. Drug Metab. Toxicol. 1, 159–168. 10.1517/17425255.1.1.159 16922658

[B39] Willmann S. Lippert J. Sevestre M. Solodenko J. Fois F. Schmitt W. (2003). PK-sim: a Physiologically Based Pharmacokinetic 'whole-Body' Model. BIOSILICO 1, 121–124. 10.1016/s1478-5382(03)02342-4

[B40] World Health Organization (2005). International Programme on Chemical Safety (WHO/IPCS) Chemical-specific Adjustment Factors for Interspecies Differences and Human Variability: Guidance Document for Use of Data in Dose/Concentration Assessment. IPCS Harmonization Project Document No. 2. Geneva: World Health Organization, International Programme on Chemical Safety. Available at: http://whqlibdoc.who.int/publications/2005/9241546786_eng.pdf (accessed 9 27, 2021).

[B41] Yoon M. Campbell J. L. Andersen M. E. Clewell H. J. (2012). Quantitative *In Vitro* to *In Vivo* Extrapolation of Cell-Based Toxicity Assay Results. Crit. Rev. Toxicol. 42, 633–652. 10.3109/10408444.2012.692115 22667820

[B42] Zhuang X. Lu C. (2016). PBPK Modeling and Simulation in Drug Research and Development. Acta Pharm. Sin B 6, 430–440. 10.1016/j.apsb.2016.04.004 27909650 PMC5125732

