# Supplementary material for: Use of Physiologically-Based Kinetics Modelling to Reliably Predict Internal Concentrations of the UV Filter, Homosalate, After Repeated Oral and Topical Application
Source: Front Pharmacol. 2022 Jan 4;12:802514. doi: 10.3389/fphar.2021.802514 (PMC8763688; doi:10.3389/fphar.2021.802514)
Supplement: Supplementary file 1 [file DataSheet1.docx]

**Supplementary Materials**

***In silico* predictions of the potential for homosalate to be a transporter substrate**

**ADMET Predictor 9.5**

| Pgp_Substr | Pgp_Inh | OATP1B1_Inh | OCT2_Inh | BSEP_Inh | BCRP_Substr |
| --- | --- | --- | --- | --- | --- |
| Predicts whether or not the compound is P-glycoprotein substrate (Yes/No). | Predicts whether or not the compound is P-glycoprotein inhibitor (Yes/No). | Predicts whether or not the compound can inhibit OATP1B1 transporter. | Predicts whether or not the compound is organic cation transporter 2 (OCT2) inhibitor. | Predicts whether or not the compound is bile salt export pump (BSEP) inhibitor. | Predicts whether or not the compound is breast cancer resistance protein (BCRP) substrate (Yes/No). |
| No (81%) | No (96%) | No (76%) | No (69%) | Yes (83%) | No (95%) |

**SwissADME**

Second substrate classification model provided and trained on 1033 molecules by the Swiss Institute of Bioinformatics validated by an external test set (accuracy of 89%) was applied according to Daina et al. ^1^. Homosalate is in the applicability domain and was predicted as a non-substrate of P-gp.

**References**

1. Daina A, Michielin O, Zoete V. SwissADME: a free web tool to evaluate pharmacokinetics, drug-likeness and medicinal chemistry friendliness of small molecules. *Sci Rep*. 2017;7:42717.

**Input parameters for the Mobi dermal model**. Physicochemical properties of homosalate were taken from the REACH dossier (ECHA 2020)].

| **Input parameter** | **Value** |
| --- | --- |
| Log P_OW_ | 6.34 at 40°C |
| Boiling point | 295.1°C at 101.3 kPa |
| Melting point | < -20°C at 101.3 kPa |
| Vapor pressure | 0.015 Pa at 25 °C |
| Molecular weight | 262.344 |
| Water solubility | 0.4 mg/l at 25 °C |
| pKa | 8.1 ± 0.3 at 20 °C |
| Relative density at 20°C relative to water at 4°C | 1.0512 (1.050-1.053) |
| Grain class | Organic compound |
| Pharmacophore | False (neutral compound) |
| Model type | *In vivo* |
| Skin hydration | Full |
| Skin layer thickness | Stratum corium: 43µm  Epidermis: 40µm  Dermis: 1400µm |
| Skin Surface Temperature | 30°C |
| Volatile | False |
| Wind Velocity | 16.8 cm/s |
